# Metagenomics analysis reveals features unique to Indian distal gut microbiota

**DOI:** 10.1371/journal.pone.0231197

**Published:** 2020-04-08

**Authors:** Kamaldeep Kaur, Indu Khatri, Akil Akhtar, Srikrishna Subramanian, T. N. C. Ramya

**Affiliations:** Department of Protein Science and Engineering, CSIR-Institute of Microbial Technology, Chandigarh, India; University of Minnesota Twin Cities, MN, UNITED STATES

## Abstract

Various factors including diet, age, geography, culture and socio-economic status have a role in determining the composition of the human gut microbiota. The human gut microbial composition is known to be altered in disease conditions. Considering the important role of the gut microbiome in maintaining homeostasis and overall health, it is important to understand the microbial diversity and the functional metagenome of the healthy gut. Here, we characterized the microbiota of 31 fecal samples from healthy individuals of Indian ethnic tribes from Ladakh, Jaisalmer and Khargone by shotgun metagenomic sequencing. Sequence analysis revealed that *Bifidobacterium* and *Prevotella* were the key microbes contributing to the differences among Jaisalmer, Khargone and Ladakh samples at the genus level. Our correlation network study identified carbohydrate-active enzymes and carbohydrate binding proteins that are associated with specific genera in the different Indian geographical regions studied. Network analysis of carbohydrate-active enzymes and genus abundance revealed that the presence of different carbohydrate-active enzymes is driven by differential abundance of genera. The correlation networks were different in the different geographical regions, and these interactions suggest the role of less abundant genera in shaping the gut environment. We compared our data with samples from different countries and found significant differences in taxonomic composition and abundance of carbohydrate-active enzymes in the gut microbiota as compared to the other countries.

## Introduction

The human gastrointestinal tract harbors an extremely large microbial community including archaea, bacteria, viruses and eukaryotes. These communities are very complex and dynamic in humans, varying with age [1], diet [2], genetics [3,4], geography, disease [5–8], infection and other factors [9]. Around 10^13^–10^14^ bacteria inhabit the human body and most of them reside in the colon [10]. Microbes are initially acquired from the mother and the immediate environment during birth [11,12] and they further co-evolve within the human host as per several factors as stated above. The gut microbiota are thought to play an important role in immunomodulation [13], defense against pathogens [14], nutrient and energy harvest, and metabolism [15–18]. Considering the important role of the gut microbiome in maintaining homeostasis, it is important to understand the microbial diversity and the functional metagenome of the healthy gut.

Despite the fact that significant intra- and inter-individual variations exist in the taxonomic composition of microbial communities in the human distal gut, there are several initiatives aiming to find common patterns among two or more different groups of interest. Identifying the differences and similarities among the healthy gut microbiota between two geographies has been important to understand the impact of the environment and diet. With this aim, several metagenomics initiatives have been taken in several regions across the world such as China [7,18], Russia [19], Europe [20,21], USA [22], Venezuela [1], Africa [23], Ireland [2], Italy [24], Japan [25] and Korea [26].

Several studies have previously been performed on Indian subjects, too, with their wide focus on healthy and malnourished children [27,28], lean, obese and surgically treated obese individuals of Indian origin [29], Mongoloid and Proto-Australoid tribal populations from North-East India and South India [30], healthy individuals from urban and rural areas of Delhi and Pune [31], healthy individuals from high altitude and low altitude regions [32], healthy individuals from Western India [33], Bhopal and Kerala [34] and tuberculosis patients from Delhi [35]. Albeit some of these reported studies of the Indian gut microbiota are metagenomics studies [27,28,34,35], most of them are taxonomic profiling studies performed using 16S rRNA gene sequencing. These studies indicate the presence of the phyla, Firmicutes, Bacteroidetes, Actinobacteria, Proteobacteria, Spirochetes, Verrucomicrobia and Fusobacteria, and the genera, *Prevotella*, *Megasphaera*, *Faecalibacterium*, *Eubacterium*, *Clostridium*, *Blautia*, *Collinsella*, *Ruminococcus*, *Roseburia*, *Bifidobacterium*, *Gordonibacter*, *Slackia*, *Bacteroides*, *Odoribacter*, *Parabacteroides*, *Clostridium*, *Enterobacter*, *Escherichia*, *Vibrio*, *Pseudomonas*, *Klebsiella* and *Pantoea* in the gut microbiome of Indian subjects [30–32,34]. They further suggest that distinct taxonomic profiles might be present in subjects from different geographic regions and dietary habits. For instance, *Prevotella* was found to be the dominant genus in North-Central India (where subjects mostly consumed a plant-based diet), whereas *Bacteroides*, *Ruminococcus* and *Faecalibacterium* were prominent in the gut microbiome of the cohort from Southern India (with a more omnivorous diet) [34]. Thus, existing studies of Indian subjects provide taxonomic profiles of the gut microbiota, i.e., information of the presence or absence of various microbial taxa in the individual samples, and hint at the potential diversity of gut microbiota composition in different regions of India. However, a survey of the literature in this field also indicates a lack of metagenomic sequence data that can facilitate analysis of functional profiles of the microbiota in Indian subjects.

The gut is a carbohydrate-rich niche containing diverse carbohydrates that include plant polysaccharides such as xylan and starch (from dietary plant products), animal tissue polysaccharides such as chondroitin sulphate (from dietary meat), milk oligosaccharides, mucus glycans, and microbial saccharides such as levan and dextran [36]. The human gut microbial metagenome contains tens of thousands of genes encoding carbohydrate-active enzymes (CAZymes) involved in the metabolism of various carbohydrates as compared to just eight host enzymes known to digest carbohydrate nutrients in the gut [37]. Microbial CAZymes and carbohydrate-binding proteins play a critical role in the successful colonization of the gut (withstanding peristalsis and rapid rate of turnover of host epithelial cells) by enabling the retention of microbes within the gut via adhesion to host glycans [38–41], and by facilitating utilization of the diverse carbohydrates present in the gut milieu. Thus, diets varying in their carbohydrate component are expected to be utilized by different sets of microbial CAZymes and thus select for different resident microbes.

We thus framed our study with the following considerations–one, the important role of the gut microbes for homeostasis and health, two, reported differences among the healthy gut microbiota in subjects from different geographies and diet, and three, the importance of microbial CAZymes and carbohydrate-binding proteins in determining colonization and utilization of dietary carbohydrates. Our aim was therefore to study the taxonomic composition and diversity of CAZymes in healthy subjects and identify the key microbes that contribute to varying taxonomic composition and CAZyme diversity among healthy subjects from different geographies and diets, both within and without India.

Considering the dearth of publicly available metagenomic sequence data for Indian subjects, we initiated our study with deep metagenomic sequencing of the distal gut microbiota of healthy subjects. Taking into account the prevalence of diverse ethnicities, environmental conditions, cuisines and cultures in India, we selected for this study healthy adult subjects from three different rural geographical locations (in the Central, Northern and Western regions of India), selected for their distinct geographic and climatic conditions as well as ethnic origins and dietary habits of the resident subjects. In addition to analyzing the gut metagenomes of these three geographic regions of India, we performed comparative analysis of the Indian gut metagenomes with gut metagenomes of subjects from other countries viz. Germany, USA, Denmark, France and China in order to reveal differences in taxonomic composition and diversity of carbohydrate active enzymes (CAZymes) in these subjects of different nationalities. This study thus reveals biologically relevant differences in the taxonomic structure and carbohydrate-active enzyme diversity of human gut microbiota across different geographies.

## Material and methods

### Ethics approval and consent

This study was approved and conducted in accordance with guidelines laid by Council of Scientific and Industrial Research (CSIR)- Institute of Microbial Technology Institutional Ethics Committee (Human) (Project number 11 IEC/1/9-2014) and Council of Scientific and Industrial Research (CSIR)- Institute of Microbial Technology Institutional Biosafety Committee (Project number IBSC/2012-2/21). Written informed consent was obtained to release the information obtained as a result of the subject’s participation and to publish the study while keeping the identity confidential.

### Sample collection

Subjects included for the study were self-reported healthy individuals with local dietary intake. The exclusion criteria were antibiotic intake in the six months prior to sample collection, and major surgeries of the gastrointestinal tract. Fecal samples were collected from thirty-one healthy subjects, in the age group of 18–58 years, from different regions of India viz. Ladakh (34.425960°N 76.824421°E), Khargone (22.226704° N, 75.863329° E), and Jaisalmer (26.36539° N, 70.42584° E). Samples from Ladakh, Jaisalmer, and Khargone were collected on 27 October 2013, 9 November 2014, and 25–26 November 2014, respectively. Subjects participating in the study were instructed to hand over fecal samples in a sterile container within 30 minutes of defecation. In Jaisalmer and Khargone, fecal samples were stored on dry ice soon after collection from the subjects and transported on dry ice to our laboratory in CSIR-IMTECH, Chandigarh. In Ladakh, fecal samples were collected on ice packs and transported to a -80°C freezer in Defence Institute of High Altitude Research (DIHAR), Leh, Ladakh (in ~4 hours). From there, the samples were transported on dry ice to our laboratory in CSIR-IMTECH. Upon reaching our laboratory, samples were stored in a -80°C freezer until DNA extraction. The metadata of the nine subjects from Ladakh, 12 subjects from Khargone, and 10 subjects from Jaisalmer are provided in **S1 Table**.

### Whole genomic DNA isolation

Metagenomic DNA was isolated from the fecal samples using ZR Fecal DNA MiniPrep^™^. Up to 150 mg of each frozen fecal sample was scraped off with the help of a sterilized spatula (to avoid freeze thaw and microbial contamination) and transferred into a tube provided with the kit. After addition of lysis buffer, samples were homogenized by FastPrep 120 at speed 6 m/s for 40 seconds followed by cooling on ice for 1 minute, thrice. The rest of the protocol was performed as per the instructions in the user manual. This kit does not contain any RNAse step, so the nucleic acids isolated by the ZR kit were treated with 10 μl of RNAse (10 mg/ml stock) for 30 minutes at 37°C to remove any contaminating RNA. Following this, the sample was subjected to phenol/Sevag extraction and the nucleic acids recovered from the aqueous phase by ethanol precipitation and suspended in TE. DNA quality was checked by visual examination of the DNA upon gel electrophoresis on 1% agarose and DNA quantitation was performed using NanoDrop 1000 spectrophotometer.

### Metagenome sequencing

Genome sequencing of all the samples was performed on the Illumina HiSeq-2500 platform. Jaisalmer samples and Khargone samples were sequenced at 2*150 bp reads using Illumina HiSeq-2500 sequencing technology at Genome Quebec Centre (GQC), McGill University, Montreal, Canada. Ladakh samples were sequenced at 2*151 bp reads using Illumina HiSeq-2500 sequencing technology at Macrogen, Korea. Library preparation for all samples was carried out according to the Nextera XT sample preparation protocol (Illumina, Inc., San Diego, CA).

### Preprocessing of reads

The shotgun data obtained for all the samples were assessed for their quality using FASTQC (http://www.bioinformatics.babraham.ac.uk/projects/fastqc). Identified ‘Nextera transposases’ and ‘Illumina universal’ adaptor sequences were removed using the ‘Trim Sequences’ module of CLC genomics workbench (www.clcbio.com). Further, the reads obtained were filtered for the presence of human contamination using DeconSeq [42] at 90% coverage and 95% identity. Low-quality reads below Phred Score 20 and length less than 50 bases were filtered out using NGS QC Toolkit [43].

### Taxonomic profiling of reads

High quality reads obtained after the preprocessing of the reads were analyzed for microbial composition using MetaPhlAn2 [44] and OneCodex [45]. MetaPhlAn relies on unique clade-specific marker genes identified from 3,000 reference genomes whereas One Codex identifies microbial sequences using a "k-mer based" taxonomic classification algorithm, using a reference database that includes approximately 40,000 bacterial, viral, fungal, and protozoan genomes. We applied two different strategies to visualize the differences in the analysis methods of taxonomic profiling. MetaPhlan yields the organismal relative abundance whereas OneCodex yields absolute abundance of the reads classified. To compare the methods, the classified reads of each taxon from OneCodex were normalized to the total classified reads at that taxonomic level of the sample. To assess if the reads depict the overall diversity of the taxonomically classified taxa, a rarefaction curve was drawn using MEGAN [46,47]. This curve is a plot of the number of taxa assigned (as per MEGAN algorithm) as a function of the number of reads for each sample. It represents the richness of metagenomic datasets and also tells if sequencing depth is enough to capture all the diversity. Shanon Index [48] was calculated to obtain the diversity profiles of the samples.

### Assembly and annotation

High-quality reads, obtained after pre-processing, were subjected to *de novo* assembly using IDBA-UD [49] (an iterative *De Bruijn* Graph De Novo Assembler) at iterated value of K from 20 to 120 to get final longer confidently assembled contigs. The contigs longer than 500 bp were retained and subjected to MetaGeneMark [50] for gene prediction. The genes ≥ 100 bp were retained for all the samples and the corresponding protein sequences were retrieved. A non-redundant gene set for each sample was constructed with CD-HIT [51] at 95% sequence identity and 90% alignment coverage for the shorter sequence.

### Abundance and diversity of CAZymes in the gut metagenomes

The detection of the CAZymes in the assembled and annotated protein sequences from each metagenome sample was performed using HMM database for automated carbohydrate-active enzyme annotation (dbCAN) [52] latest version: 6.0 released on 09/13/2017. HMMscan was run against the dbCAN database and the alignment results were parsed through the HMMscan-parser.sh, provided by dbCAN, and the best-hit alignment was retained. HMMScan profiles for CAZyme families belonging to the classes of Glycosyl hydrolases (GH), Polysaccharide lyases (PL), Glycosyltransferases (GT) and Carbohydrate Esterases (CE), Carbohydrate Binding Modules (CBM) and other auxiliary enzymes (AA) were used to profile the CAZymes.

Extensive parameter exploration was done to ensure that the maximum number of homologs of CAZymes is detected in hitherto uncharacterized gut microbiota and yet stringency is maintained to avoid false positives (E-value < 1e -05 and query coverage > = 70%)

Each HMM hit was tagged to particular CAZyme family (GH1, GH2 etc.) and the number of hits belonging to each CAZyme family for each metagenome was collated and this was subsequently represented as a matrix, termed as the 'abundance profile'. The abundance profile for each metagenome was subsequently normalized by the total number of genes. This was done to even out the heterogeneity arising for differential metagenomic sampling for individual dataset. To the 'diversity' of a metagenomic sample, the total number of CAZyme families to which a hit was tagged in a metagenome was calculated. However, if the number of hits belonging to one particular family was less than 0.01% of the total number of hits, that family was not considered while calculating 'diversity'. To identify the differences between different geographies the abundance profiles were transformed to Z-score. Z-score is a standardized score and is calculated as ((‘score’-‘mean score’) / ‘standard deviation of score’). To identify the variability between families, the Welch t-test was performed. Kruskal-Wallis test was used for the multiple-group testing. The abundance with Bonferroni corrected P value (<0.05) were considered to be important for the specific geography.

### Correlation of CAZymes and abundant genus

The correlation of the significant CAZymes identified with P value <0.05 in the different regions of India with the genus abundance was identified using the Pearson Correlation. The positively correlated (>0.85 correlation) CAZymes and genera were obtained and the co-occurrence was visualized in Gephi [53]. The correlations within CAZymes or genera were not visualized and only correlations between CAZYmes and genera were presented.

### Comparison of Indian samples with other countries shotgun data

The shotgun data (only those using Illumina 2000/2500/Genome Analyzer II/IIx) publicly available for healthy human gut microbiota samples from several other countries was retrieved. It comprises samples from the United States, Denmark, France, China and Germany. These samples were selected randomly and the raw reads were processed in a similar way as we did for Indian samples to remove the methodological bias. After assembling the reads for other countries, the samples with very few reads assembled (<50%) were removed from the CAZyme analysis. This was done to remove the samples which can skew the analysis.

### Statistical analysis

Multinom{nnet} R package was used to perform Analysis of variance (ANOVA) to find any differences at significant P values for multiple-group comparisons. The two group comparisons were performed using Welch t-test and Bonferroni corrected P values are indicated for each comparison. Shannon diversity is calculated using vegdist{vegan} function in R-statistical tool. Partial Least Squares discriminant analysis (PLSDA) was used for the unsupervised clustering of the samples. Heatmaps were generated based on the dissimilarity matrix of the Shannon diversity, which indicates that higher score will indicate lower similarity and vice-versa.

## Results

### Subject selection

**Fig 1:**
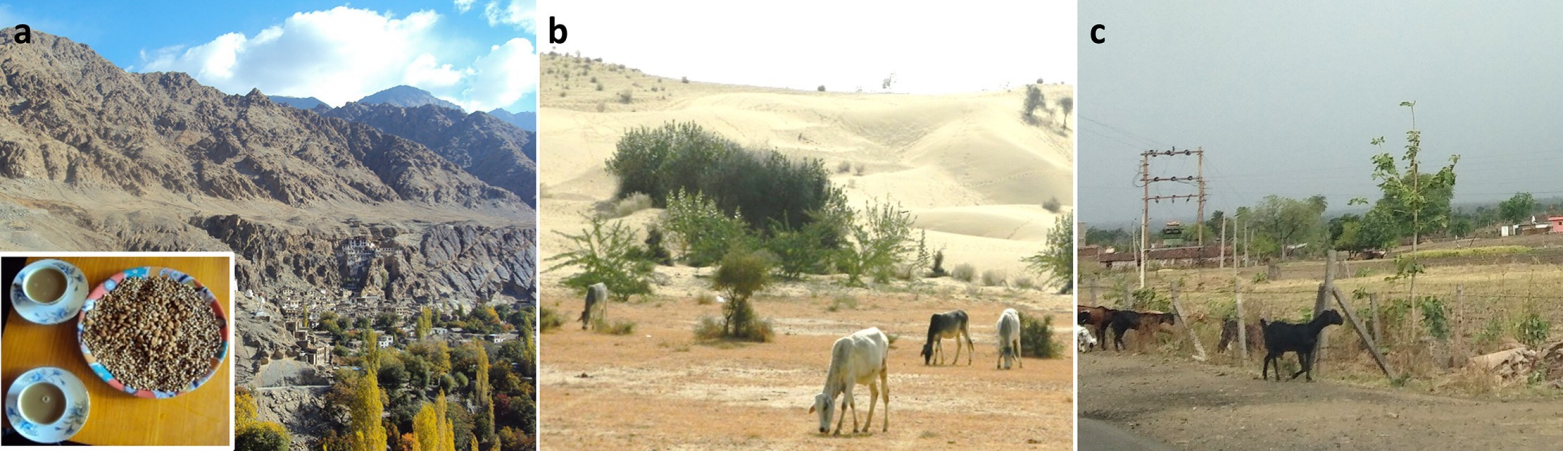
Sampling sites of this study. a) Photographs showing the sampling site, and typical food consumed (gurgur cha—tea with yak butter and salt, and tsampa—roasted barley flour) in Scur Buchan village in Ladakh, Jammu and Kashmir (34.425960°N 76.824421°E). b) Photograph showing the sampling site in Khuri village in Jaisalmer district, Rajasthan (26.36539° N, 70.42584° E). **c**) Photograph showing the sampling site in Pipliya Buzurg village in Khargone district, Madhya Pradesh (22.226704° N, 75.863329° E).

We used 31 fecal samples of healthy adult subjects from three geographical locations in India for studying the gut microbial community structure (**Fig 1**). The sampling sites were relatively less populated settlements of ethnic Indians in three rural areas–Scur Buchan village in Ladakh, Jammu and Kashmir (34.425960°N 76.824421°E), Pipliya Buzurg village in Khargone district, Madhya Pradesh (22.226704° N, 75.863329° E), and Khuri village in Jaisalmer district, Rajasthan (26.36539° N, 70.42584° E), respectively. The geographic and climatic conditions of these three sampling sites are distinct, as are the ethnic origins and dietary habits of the subjects from these sites (**Table 1**).

**Table 1:** Sampling sites of this study.

Site	Geography	Climate	Predominant race	Major cuisine
**Ladakh**	Extends from the Himalayas in the South to the Kunlun range in the North. Includes the upper Indus River valley Elevation: >3,000 m	Cold desert	Mongoloid	*Momos*, *thukpa* and *thenthuk* (noodle soups), *khambir* (whole wheat bread), *gurgur cha* (tea with yak butter and salt), *tsampa* (roasted barley flour) and *chhang* (an alcoholic beverage)
**Jaisalmer**	Located in the Thar desert Elevation: 225 m approx.	Hot arid and semi-arid	Caucasoid	*Roti (whole wheat bread)*, spicy curries, *dal* (lentil soup) and *chhaach* (butter milk)
**Khargone**	Situated in the middle of the Narmada River valley; Vindhyachal mountain range is situated in the north and Satpura range in the south Elevation: 258 m approx.	Transitional between humid subtropical climate and tropical wet and dry climate	Australoid	*Dal (lentil soup)*, *chawal (boiled rice)*, *roti (whole wheat bread)*, *sabzi (vegetables in curry or fried)*, tea, and milk

Geographical features, climatic conditions, racial features, and major cuisines of the three regions of India selected for this study.

We included 9 subjects (7 male, 2 female) aged 39.44 ± 3.56 (mean ± SEM) with a body mass index (BMI) of 23.98 ± 0.97 (mean ± SEM) from Ladakh, 12 subjects (all male) aged 30.08 ± 2.00 (mean ± SEM) with a BMI of 22.63 ± 0.97 from Khargone, and 10 subjects (8 male, 2 female) aged 32.9 ± 4.49 years (mean ± SEM) with a BMI of 20.73 ± 0.83 (mean ± SEM) from Jaisalmer. On the whole, the average age of all the subjects was 33.71 ± 2.00 (mean ± SEM) and the average BMI was 22.41 ± 0.57 (mean ± SEM). The basic metadata of all the samples is provided in **S1 Table**. Eleven of twelve Khargone subjects, nine of ten Jaisalmer subjects and all nine Ladakh subjects consumed dairy products (**S1 Table**). Khargone subjects included three vegetarian, one ovo-vegetarian, and eight non-vegetarians with occasional poultry/red meat/fish in the diet (**S1 Table**). Jaisalmer subjects comprised four vegetarian and six non-vegetarians with occasional red meat/poultry in the diet (**S1 Table**). Ladakh subjects included two ovo-vegetarian and seven non-vegetarians with occasional poultry/red meat/fish in the diet (**S1 Table**). The detailed diet information for each participant is mentioned in **S2 Table**. None of the subjects reported antibiotic use in the last six months.

We isolated metagenomic DNA from the fecal samples and subjected the metagenomic DNA to shot gun sequencing on the Illumina platform. We obtained 8.59 ± 0.38 (mean ± SEM) Gbp data per sample. Following the steps of filtering to remove adapter sequences and human contamination, and quality trimming of the reads, 89.88 ± 0.41% data (i.e., 7.73 ± 0.35 Gbp) remained (**S3 Table**). The GC content was 47.59 ± 0.35% (**S3 Table**).

### OneCodex and MetaPhlAn based comparative taxonomic characterization of the gut microbiota in Indian subjects

The taxonomic structure of the microbiota in the cohorts from Ladakh, Khargone and Jaisalmer using OneCodex reveal that less than half of the reads could be mapped at different taxonomic levels (phylum level: 45.27% ± 1.82; family level: 42.94% ± 1.77; genus level: 41.83% ± 1.56, Mean % ± SEM) (**S3 Table**). Lower read mapping (~55%) at the phylum level indicates that many gut bacterial species are yet to be characterized. However, by plotting a rarefaction curve, we found that the depth of reads sequenced is sufficient to enrich for all presently known genera (**S1 Fig**). We obtained similar genus abundance profiles with OneCodex [45] and MetaPhlan as verified by PLSDA and visualized by heat maps (**S2 Fig**). Box plots of family and genus abundances also indicate similar profiles (**S3 Fig**).

We identified a total of 61 phyla, 93 classes, 232 orders, 530 families, and 2052 genera of microorganisms by One Codex, and a total of 9 phyla, 16 classes, 21 orders, 44 families, and 88 genera of microorganisms by MetaPhlAn in the Indian subjects. The major phyla present in the Indian subjects, together constituting more than 90% of microbiota in all the samples are tabulated (**Table 2, S4 Table**). The most abundant families and genera identified in the Indian subjects by One Codex and MetaPhlAn analysis were almost similar (**Table 2, S3 Fig and S4 Table**).

**Table 2:** Taxonomic profiling of gut microbiota in Indian subjects by OneCodex and MetaPhlAn.

		Total Gene Abundance	
		OneCodex (%)	MetaPhlAn (%)
**Phyla**	Bacteroidetes	53	41
	Firmicutes	31	38
	Actinobacteria	12	17
	Protobacteria	3	2
**Family**	Prevotellaceae	47	39
	Bifidobacteriaceae	10	15
	Ruminococcaceae	8	9
	Lachnospiraceae	7	6
	Bacteroidaceae	6	1
	Eubacteriaceae	4	7
	Lactobacillaceae	3	6
	Veillonellaceae	4	6
**Genus**	*Prevotella*	47	39
	*Bifidobacterium*	11	15
	*Faecalibacterium*	6	6
	*Bacteroides*	6	1
	*Lactobacillus*	3	6
	*Eubacterium*	4	7
	*Ruminococcus*	2	3

Total gene abundance at the phyla, family and genus level in Indian subjects as identified by OneCodex and MetaPhlAn. The data is supported by Supplementary information in **S4 Table**, **S2 Fig** and **S3 Fig**.

### Existing variations in the gut microbiota of Indian subjects

**Fig 2:**
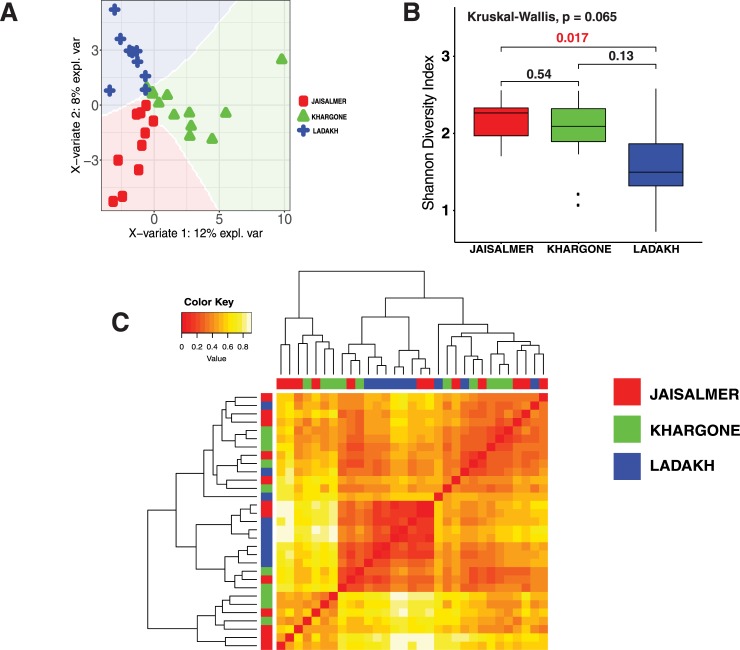
Indian gut taxonomic structure. a) Differences in the microbial relative abundance at the genus level of human gut in Indian regions. b) Shannon diversity indices of genus abundance in different regions of India. The multiple-group comparison is made using Kruskal-Wallis test and the P value for the testing is mentioned in the top-left of the plot. Welch t-test Bonferroni corrected P values for pairwise comparisons are also shown in the plot. Values indicated in red are significant. **c)** Dissimilarity matrix visualized in form of heat map generated by distance between genus abundance structure between different regions of India. Clustering of the different samples is based on the distance matrix calculated using Bray-curtis method implemented in vegdist{vegan} function in R. The lower the expression value (indicated in key) the higher the similarity.

The MetaPhlAn based taxonomic characterization was used further to identify the existing variations in Indian subjects from different geographic locations. Partial Least Squares discriminant analysis (PLSDA) of the genera abundant in Indian samples (**Fig 2A**) revealed that the structure of the intestinal microbiota in Khargone, Jaisalmer and Ladakh are different from each other. We calculated the Shannon index of bacterial alpha diversity [48] for Jaisalmer and Khargone gut microbiota to be greater than that for Ladakh gut microbiota (**Fig 2B**). We observed that the Jaisalmer and Khargone samples were interspersed amongst each other and contained similar genera with significant abundance (**Fig 2C**). In contrast, the Ladakh samples mostly clustered separately (**Fig 2C**).

*Prevotella*, the most abundant genus among all Indian subjects (**S3** and **S4** Figs and **S4 Table**), was significantly more abundant in Ladakh samples (~63%; ~58%) than in Jaisalmer (~42%; ~30%) and Khargone samples (~41%; ~32%) by One Codex and MetaPhlAn analysis (**S3** and **S4** Figs and **S4 Table**). In contrast, *Bifidobacterium*, the second most abundant genus among all Indian subjects (**S3 Fig**), was present at greater abundance in Jaisalmer (~13%; ~21%) and Khargone (~13%; ~17%) as compared to Ladakh (~4%; ~6%) (**S4 Fig**). We could also observe differences in the less abundant genera between the samples from these different geographic regions of India (**S5.1 Table**).

At the phylum level, we observed that subjects from Ladakh had a much higher proportion of microbes belonging to phylum Bacteroidetes (~70%; ~62%) as compared to Khargone (~46%; ~35%) and Jaisalmer (~47%; ~31%) by One Codex and MetaPhlAn analysis (**S3** and **S4** Figs). In contrast, Jaisalmer and Khargone subjects had a higher proportion of Actinobacteria (Jaisalmer: ~15%; ~23%, Khargone: ~14%; ~19% and Ladakh: ~6%; ~7%) and Firmicutes (Jaisalmer: ~36%; ~43%, Khargone: ~35%; ~42% and Ladakh: ~21%; ~28%) (percent values by One Codex and MetaPhlAn analysis) (**S3** and **S4** Figs).

### Comparing the microbial community structure of Indian gut with other countries

**Fig 3:**
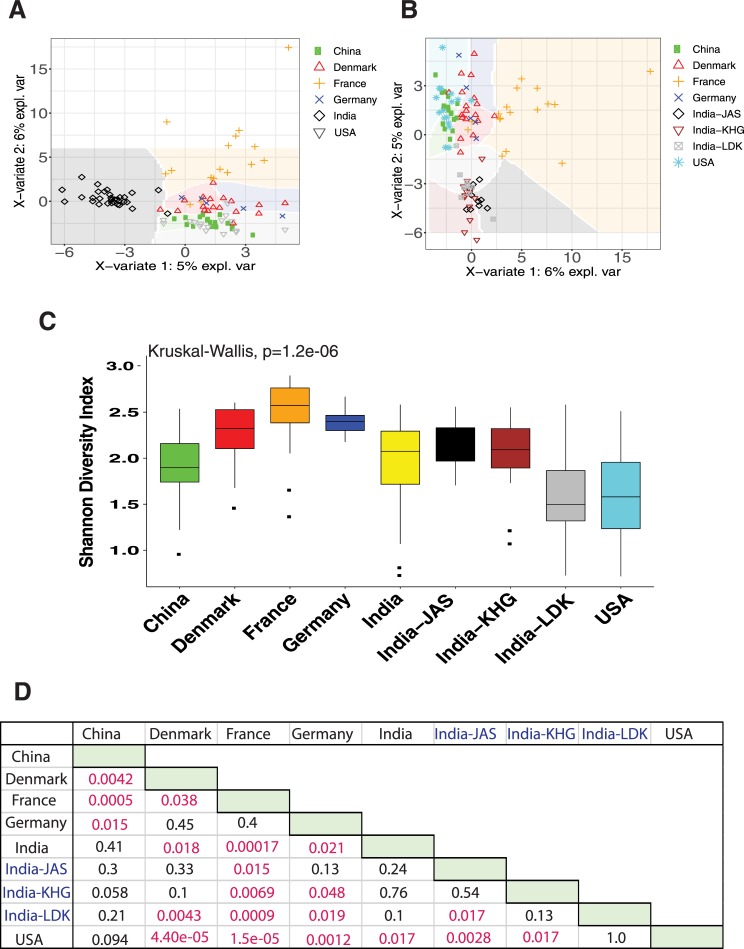
World gut taxonomic structure in comparison to Indian gut. a) Differences in microbial relative abundance at the genus level of human gut in different countries. All Indian samples were pooled together for this analysis. b) Differences in microbial relative abundance at the genus level of human gut in different countries. Samples from Jaisalmer, Khargone and Ladakh were used separately for this analysis. c) Shannon diversity indices of the genus abundance in different countries. ‘India’ refers to all samples from Jaisalmer, Khargone and Ladakh pooled together. The multiple-group comparison is made using Kruskal-Wallis test and the P value for the testing is mentioned in the top-left of the plot. d) Welch t-test Bonferroni corrected P values for pairwise comparisons are also shown. The values indicated in red are significant. This data is supported by supplementary information in **S4, S5** and **S7** Tables.

We compared the microbiota composition of the Indian subjects in this study with existing metagenomic shotgun sequencing data sets from healthy adult subjects of US (n = 16, 10 male and 6 female), Denmark (n = 18, 9 male and 9 female), France (n = 17, 9 male and 8 female), China (n = 18, 10 male and 8 female), and Germany (n = 5, all male) (**S5.2, S6** and **S7** Tables). The community structure of Indian gut was distinct from the other countries as represented in the heat map (**S5 Fig**). Unsupervised clustering of all the samples shows clear separation of Indian samples from those of other countries (**Fig 3A**). Clustering of Indian samples was observed regardless of whether samples from Jaisalmer, Khargone and Ladakh were pooled together or not (**Fig 3A and 3B**). To find the clear patterns of community diversity at genus level we looked into Shannon diversity of all the countries. The subjects from Ladakh and USA had very low diversity at the genus level (with one particular community most abundant) as compared to other countries (**Fig 3C**). Subjects from France, Denmark, and Germany displayed higher diversity than Khargone and Jaisalmer samples (**Fig 3C**).

Microbial species belonging to the well represented genera, *Lactobacillus*, *Prevotella* (in Ladakh subjects), and *Bifidobacterium* (in Khargone and Jaisalmer subjects) were found be more abundant in Indian subjects than in subjects from other countries (**S5 Fig**); whereas *Bacteroides* was more abundant in other countries as compared to the Indian subjects (**S5 Fig**). Similar variations were observed in other genera of lower abundance as well, which could clearly separate the Indian gut from the gut belonging to other countries in terms of microbial composition (**S5.2 Table**).

### CAZyme profile in the Indian gut

**Fig 4:**
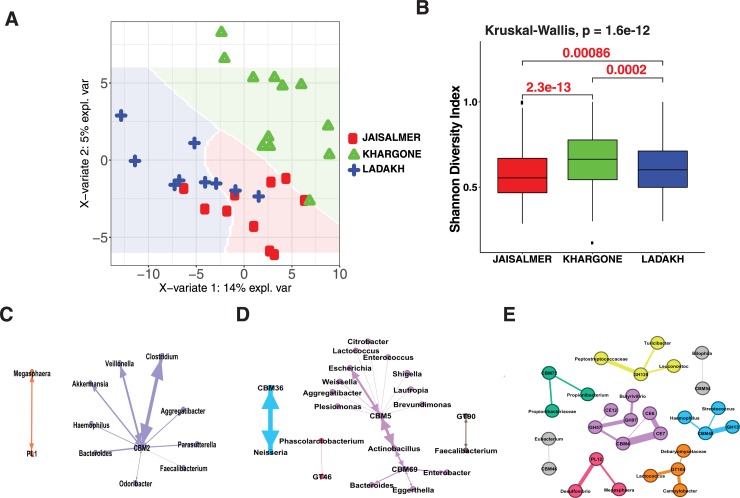
Indian gut CAZyme profile. a) The differences in the relative abundance of CAZymes in human gut in Indian regions. b) Shannon diversity indices of the CAZyme abundance in different regions of India. The multiple-group comparison is made using Kruskal-Wallis test and the P value for the testing is mentioned in the top-left of the plot. Welch t-test Bonferroni corrected P values for pairwise comparisons. The values indicated in red are significant. c,d,e) The positive correlation of CAZymes with abundant genera in different regions of India (c: Jaisalmer, d: Khargone, e: Ladakh).

A PLSDA plot of CAZyme abundance (CAZyme abundance here is relative to total bacterial genes) in the gut microbiota of subjects from different regions of India revealed differences in CAZyme abundances (**Fig 4A** and **S8 Table**). A boxplot representing the CAZyme abundance also reveals significant differences in CAZyme abundance between subjects of Khargone and Jaisalmer (**Fig 4B**). Jaisalmer samples have 10 significantly different CAZymes (*p*>0.05) in comparison to other Indian regions (**S9.1 Table**). The significantly different CAZymes in the Jaisalmer samples were mostly found to be carbohydrate binding modules (CBMs); there were no glycoside hydrolases (GHs) and carbohydrate esterases (CEs) (**S9.1 Table**). The highest number of significantly different CAZymes (30 CAZymes) was observed in Ladakh samples (as compared to 25 in Khargone and 10 in Jaisalmer) (**S9.1 Table**). The significantly different CAZymes in Khargone and Ladakh samples have representation from all the classes of CAZymes (glycosyltransferases (GTs), polysaccharide lyases (PLs), CBMs, GHs and CEs) (**S9.1 Table**).

We investigated the relation, if any, between the significantly different CAZymes obtained and genus identified for each Indian region separately (**Fig 4C, 4D and 4E**). The question we wanted to address was whether any significantly different CAZyme is correlated directly to a differentially abundant genus in that particular Indian region. In Jaisalmer samples, we found CBM2 (*p* = 0.028) to be positively correlated with several genera (**Fig 4C**). Of these genera, *Bacteroides* and *Odoribacter* are significantly different in abundance as compared to all other samples in this study (**S5.2 Table**). Similarly, in Khargone samples, CBM5 (*p* = 0.036) and CBM69 (*p* = 0.039) are positively correlated with many genera (**Fig 4D**), of which, *Bacteroides* and *Escherichia* are abundant. *Shigella*, *Lautropia*, and *Eggerthella* are differentially abundant in Khargone samples as compared to Ladakh and Jaisalmer. *Brevundimonas*, *Actinobacillus*, *Bacteroides*, and *Escherichia*, are significantly different in Khargone samples as compared to all other samples in this study including those from other countries. CBM5, CBM69 and GT46 correlated genera are not abundantly represented. These genera are albeit not abundant, significantly differentially abundant in Khargone samples as compared to Ladakh and Jaisalmer, as well as to all other countries included in this analysis. In Ladakh samples, there are several CAZymes correlated with different genera (**Fig 4E**). Relatively abundant genera, *Streptococcus* and *Haemophilus* are connected to CBM48 (*p* = 0.0025) and GH13 (*p* = 0.024). *Butyrivibrio* is positively correlated with CE12, GH57, GH97, CBM4, CE7 and CE6. These different highly correlated networks involving a few common genera demonstrate the key differences between the subjects from Ladakh, Khargone and Jaisalmer. Importantly, these interactions involve several less abundant genera that are differentially abundant among these regions rather than major enterotype genera.

### Comparing the CAZyme profile of Indian gut with other countries

**Fig 5:**
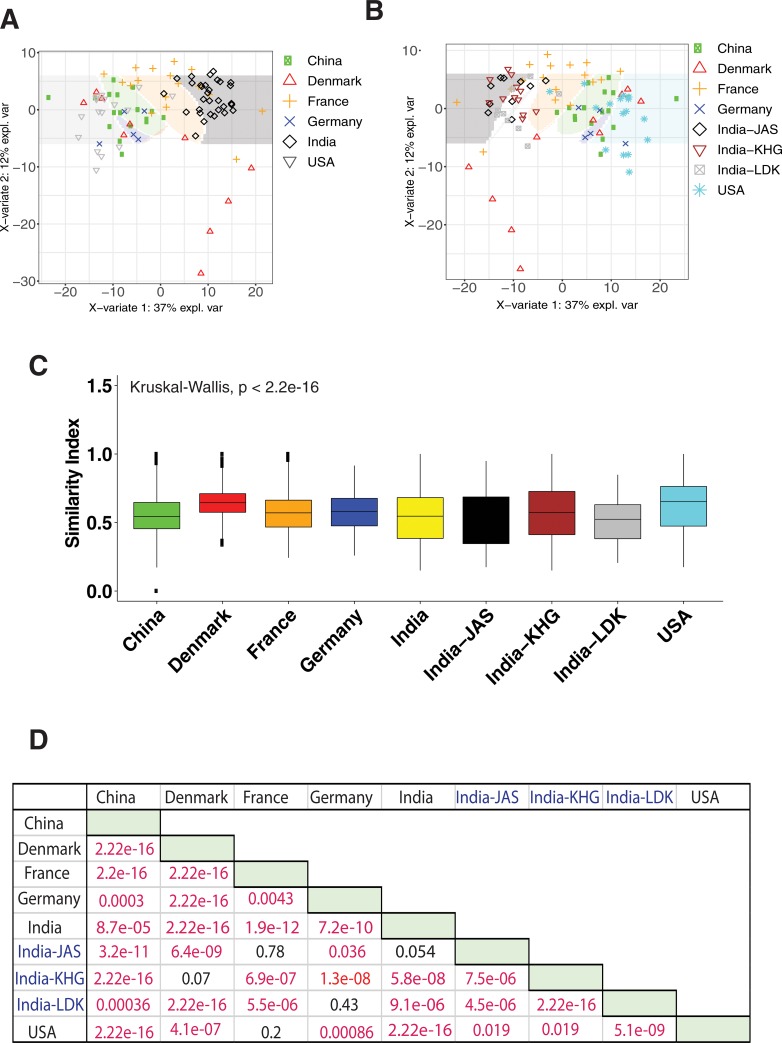
World gut CAZyme profile in comparison to Indian gut. a) Difference in the CAZyme abundance of human gut in different countries. All Indian samples were pooled together for this analysis. b) Difference in the CAZyme abundance of human gut in different countries including different Indian regions. c) Significance of CAZyme abundance as represented by Shannon diversity index in different countries. The multiple-group comparison is made using Kruskal-Wallis test and the P value for the testing is mentioned in the top-left of the plot. d) Welch t-test Bonferroni corrected P values for pairwise comparisons are mentioned in a tabular format. The values indicated in red are significant.

CAZyme profiles were retrieved from the shotgun sequencing data of other countries (**S8 Table**) and the differential abundance of CAZymes between subjects from India and other countries was studied. PLSDA plots were constructed; they clearly demonstrated the demarcation of Indian subjects from those of other countries in terms of CAZyme abundance (**Fig 5A and 5B**). The Indian regions were considered pooled together as well as separately for this analysis in order to identify the differential abundance of CAZymes in the different Indian regions as compared to the other countries (**Fig 5A and 5B**). A boxplot for CAZyme abundance was plotted and ANOVA performed to identify the level of significance as compared to all other countries (**Fig 5C**). The CAZyme abundance in Khargone and Ladakh regions (but not in Jaisalmer) was significantly different as compared to all other countries (**Fig 5C**). With further analysis we found that Jaisalmer subjects had significant differences in the abundance of various CAZymes as compared to all the countries except Germany. The number of significantly (*p*<0.05) differentially abundant CAZymes was ~170 for Jaisalmer and Khargone subjects and ~120 for Ladakh subjects. Indian samples were found to have only 2–3 uniquely differentially abundant CAZymes if compared with all the countries. However, when compared with France, Germany and Denmark, 100–120 uniquely differentially abundant CAZymes were found. This suggested that the CAZyme profiles in Indian subjects have a similar profile as that of China and USA. Among the subjects of the other countries, we found 172 significant differentially abundant CAZymes at significant P values in China samples and 8 CAZymes were unique to samples from China (**S9.2 Table**). Interestingly, there were no CE specific classes unique to Denmark and USA samples and no GT specific CAZymes that were unique to Germany samples. The differentially abundant CAZymes in other countries had overlapping profiles among them or with Indian countries. For example, GH109, GT2, CBM67, PL12, dockerin and PL11 are present in both France and USA. This suggests why there were no clear demarcations in the PLSDA plot as there are more shared CAZymes between different countries. These results imply that no unique CAZymes are present in the human gut of different countries but that their abundance is significantly different among different countries.

## Discussion

India comprises about one-sixth of the world’s population with vast geographical, linguistic, socio-cultural and ethnic diversity. Indian population is genetically heterogeneous [54] with Australoid, Caucasoid, Mongoloid and Negrito ancestries [55]. Despite genetic admixtures between diverse lineages, there is a high level of genetic differentiation amongst contemporary ethnic groups present in different regions of India [54]. The regions included in this study include Mongoloid (Ladakh), Caucasoid (Jaisalmer) and Australoid (Khargone) populations [56]. There are also vast climatic and socio-cultural differences among these places.

Using two different tools, MetaPhlan and OneCodex to analyze the taxonomic composition from the shotgun sequencing data, we found that ~55% of the sequence reads could not be mapped even at the phylum level. Analysis of the mapped reads indicated the phyla, Bacteroidetes, Firmicutes, Actinobacteria and Proteobacteria, and the genera, *Prevotella*, *Bifidobacterium*, *Bacteroides*, *Eubacterium* and *Faecalibacterium* to be abundant in Indian subjects. The dominant phylum was Bacteroidetes and the dominant genus *Prevotella* in all the Indian subjects in the study. We found significant differences in the gut microbiota composition of the subjects from Ladakh, Khargone and Jaisalmer regions. At the phylum level, Ladakh had a higher proportion of Bacteroidetes, and Jaisalmer and Khargone subjects had higher proportions of Actinobacteria and Firmicutes. At the genus level, *Prevotella* was more abundant in Ladakh subjects than in Jaisalmer and Khargone subjects, and *Bifidobacterium* was more abundant in Jaisalmer and Khargone subjects than in Ladakh subjects. However, we did not find any correlation of genus with diet, location or age between these regions by UniFrac analysis, perhaps due to the low sample number.

A limitation of our study was the under-representation of female subjects and the somewhat high variance in the age of the subjects in our study in all the three regions sampled. However, the variation in gut microbiome between populations, as a result of differences in geography, life style, diet, age, genetics and possible other factors has been reported to be more extensive than the variation between males and females [57]. Published literature regarding the effect of age on the gut microbiota indicates that whereas the gut microbiome is very dynamic with increasing microbial diversity in the early years of life, the gut microbiota in healthy adults is relatively stable [58] albeit recent studies do indicate that the gut microbiota can serve as a chronological marker [59,60].

All samples of our study were sequenced using Illumina HiSeq-2500 platform, albeit at two different sequencing centers. To our knowledge, there is no batch effect reported due to sequencing being done with the same sequencing technology at different sequencing facilities. However, we would also like to extend a cautionary note about the potential for confounding by batch effect in our analysis with our sequenced samples and more so, with sequenced samples of subjects from other countries.

Upon comparison of the gut microbiota of the Indian subjects with other countries, we found that the genera, *Lactobacillus*, *Prevotella* and *Bifidobacterium* were significantly more abundant in Indian subjects and *Bacteroides* was more abundant in subjects from other countries. Unsupervised clustering by PLSDA indicated clear separation of Indian subjects from subjects of other countries. We would like to note here that the previous metataxonomic profiling study by Bhute et al indicated *Prevotella*, *Lactobacillus*, *Lachnospira* and *Roseburia* to be overrepresented in urban and rural Indian subjects from Delhi and Pune as compared to American subjects [31].

Western microbiomes have been considered to generally have decreased alpha diversity with 15–30% fewer species than non-Western microbiomes [61,62]. In fact, the trend of decreasing microbial diversity is observed during human evolution as observed from the “clock-like divergence of microbiomes among African apes” by Moeller et al [63]. Similar loss of microbial diversity has also been observed in non-human primates upon captivity, coinciding with loss of dietary fiber [64], and “diet-induced extinctions in the gut microbiota” have been demonstrated to be amplified over generations [65]. Among various human populations, US subjects have been shown to have the least microbial diversity [1,63].

Considering the traditional and rural agrarian nature of the subjects in this study, it was surprising that the alpha diversity calculated for the microbiota of Jaisalmer, Khargone and Ladakh subjects were not very high. Indeed, the alpha diversity of the Ladakh subjects was as low as that of the US subjects included in the study. However, considering the fact that only ~45% of the sequence reads in our study could even be mapped, we anticipate that the actual alpha diversity is much higher than the values currently calculated.

The microbiota of Western individuals also typically have a greater amount of Bacteroidetes, and lower amounts of Firmicutes and Proteobacteria than healthy indigenous tribes and rural populations from developing countries [61,62]. A study of the gut microbiota of healthy adult and children subjects from rural African Malawi, rural South American Venezuela and urban US indicated reduced alpha diversity in the US subjects as compared to the Malawi and Venezuela subjects, and similar microbial communities in the Malawi and Venezuela subjects [1]. The gut microbiota of healthy Chilean subjects were shown to display slightly higher abundance of Firmicutes (~43%) than Bacteroidetes (42%) and an unusually high abundance of the phylum Verrrucomicrobia (~8.5%) [66]. A study of the gut microbiota of the Hadza hunter gatherers in Tanzania (whose diet mainly comprises fibrous tubers and meat) in comparison with healthy Italian subjects indicated high alpha diversity, abundance of Firmicutes, absence of Actinobacteria, particularly *Bifidobacterium*, and the enrichment of *Prevotella* and unclassified Bacteroidetes and Clostridiales taxa that likely help harness energy from the fibrous plant tubers in the diet of the Hadza hunter gatherers [67].

Considering these reports, our finding of Bacteroidetes dominance in the Indian subjects seems atypical but can be rationalized considering the abundance of *Prevotella*, a genus of Bacteroidetes, involved in carbohydrate metabolism. The subjects in this study consumed a diet rich in plant-based products and dairy. Khargone subjects consumed a diet rich in wheat, rice, lentils, vegetables, dairy products (cow or buffalo), with some subjects consuming meat or fish, occasionally. Jaisalmer subjects consumed a diet rich in wheat, pearl millet, lentils, vegetables, fermented milk products and milk (from camel or cow), and occasional meat (**S2 Table**). Ladakh subjects consumed a diet rich in wheat, rice, barley, lentils, plenty of vegetables, milk mainly from yak, and occasional meat (**S2 Table**). Ladakhi people also drink chhang (made by fermention of barley), and green and butter tea (**S2 Table**).

Similar exceptions of Bacteroidetes dominance are also known in literature [62]. A comparative study in children from Italy (with a typical Western diet rich in animal protein, fat, sugar and starch) and Burkina Faso in rural Africa (with a predominantly vegetarian diet rich in plant starch and fiber from millet, sorghum, legumes and vegetables, and low in fat and animal protein with occasional chicken and termites) indicated the dominance of Bacteroidetes and depletion of Firmicutes, abundance of *Prevotella* and *Xylanibacter*, and underrepresentation of Enterobacteriaceae (*Shigella* and *Escherichia*) in Burkina Faso children as compared to Italian children [23]. Also, a study of the gut microbiota of low altitude resident Matses hunter gatherers (whose diet mostly comprises gathered tubers, plantains and fish) and high altitude resident rural agrarian healthy Tunapuco subjects (whose diet mostly comprises local agricultural produce, particularly stem and root tubers, and small animals such as guinea pig, pork and lamb) in Peru in comparison with healthy urban Norman Americans indicated higher alpha diversity in Matses and Tunapuco subjects than in Norman subjects [68]. This study however also indicated the dominance of Bacteroidetes in the rural high altitude Tunapuco subjects and dominance of Firmicutes in the hunter-gatherer Matses and the urban Norman subjects [68]. A study of the gut microbiota of coexisting hunter gatherer BaAka Pygmies and agrarian Bantu tribes from Central Africa with traditional subsistence patterns indicated similar amounts of Bacteroidetes and Firmicutes in the BaAka but higher abundance of Firmicutes in the Bantu [69]. Both Baaka and Bantu had significant amounts of Cyanobacteria, Spirochaetaceae and Actinobacteria, and displayed a gradient of decreasing alpha diversity and decreasing *Prevotella*, and Clostridiaceae when compared with US Americans [69].

Traditional rural/tribal communities from geographically distant areas—Hadza, Burkina Faso, Malawians, South Africans and Venezuelan Amerindians—have also been reported to bear significant amounts of *Treponema* [67–69]. We found *Treponema* in only a few Indian subjects. *Treponema* was previously found in high abundance in some Indian tribes [30].

Despite the huge variations in ethnicities and geography among the subjects of the three regions, Ladakh, Khargone and Jaisalmer, *Prevotella* was the most abundant genus in all the three regions. This suggests the prevalence of enterotype 2 of the three enterotypes described by Arumugam et al. [70]. *Prevotella* was also reported to be the most abundant genus in the gut metagenomes of the Tibetan [71], Malawian, Amerindian [1], and West African populations [23].

A recent study by Dhakan et al [34] used multi-omics approaches to study samples from subjects of North-Central (Madhya Pradesh) and South (Kerala) India. Their study also indicated *Prevotella* as the dominant genus in North-Central India (where subjects mostly consumed a plant-based diet), whereas *Bacteroides*, *Ruminococcus* and *Faecalibacterium* were prominent in the gut microbiome of the cohort from Southern India (with a more omnivorous diet). Another recent study by Tandon et al [33] indicated the dominance of *Prevotella* in an urban cohort from Western India, too.

Bhute et al showed the presence of Firmicutes as the major phylum in Indian subjects; other major phyla included Bacteroidetes, Proteobacteria and Actinobacteria [31]. Their study also indicated that *Prevotella* and *Megasphaera w*ere dominant genera [31]. Dehingia et al reported the presence of Firmicutes, Bacteoidetes and Actinobacteria as the major phyla across Manipur, Telangana, Assam and Sikkim regions of India, with the Manipur tribes having significantly more Bacteroidetes than Firmicutes and the Sikkim tribes having more Actinobacteria in comparison to the other tribes [30]. Their study also indicated that while *Prevotella* genus accounted for ~40% of the gut bacteria, other core gut bacteria present in all the four studied geographic regions include *Faecalibacterium*, *Eubacterium*, *Clostridium*, *Blautia*, *Collinsella*, *Ruminococcus* and *Roseburia*, and varying genera include *Bifidobacterium*, *Gordonibacter*, *Slackia*, *Bacteroides*, *Odoribacter*, *Parabacteroides*, *Clostridium*, *Enterobacter*, *Escherichia*, *Klebsiella* and *Pantoea* [30]. Some of these genera have also been found to be significantly varying across geographies in our study.

However, interestingly, another recent study comparing the microbiota of healthy Indian subjects from a high altitude rural site (in Leh, Ladakh) versus low altitude rural and urban sites (in Ballabhgarh, Haryana) indicated that although *Prevotella* was dominant in Ladakh subjects, Proteobacteria members like *Vibrio* and *Pseudomonas* were highly represented in Ballabhgarh subjects [32]. Also, in contrast to our study, where Ladakh (~70%), Khargone (~46%), and Jaisalmer (~47%) subjects were dominated by phylum Bacteroidetes (and followed by Firmicutes, Actinobacteria and Proteobacteria), Das et al. found that Firmicutes (62%) was the most highly represented phylum in both Ladakh and Ballabhgarh subjects followed by Bacteroidetes (24%), Actinobacteria (5.2%), Proteobacteria, Spirochetes, Verrucomicrobia and Fusobacteria [32]. The apparently contradictory results of the Ladakh gut microbiota in these studies could be due to different experimental design; gut microbial composition was studied by Das et al [32] using 16S rRNA taxonomic profiling (and hence might have PCR-introduced bias) whereas we used OneCodex and MetaPhlan analysis of shotgun sequence data. Furthermore, as we have already noted above, ~55% of the sequence reads in our study could not be mapped even at the phylum level, thus indicating a huge proportion of “dark microbes” that are yet to be identified. We anticipate that a clearer picture will be obtained in the future with the whole genome sequencing and annotation of more microbes, especially “the unculturables”, in the publically available database.

The dominant presence of *Prevotella* in the gut microbiome has been linked in many studies with a diet rich in plant fibre and low in animal proteins [72–75], and similarly, *Bacteroides* has been demonstrated to be abundant in subjects with animal protein-rich diets [23,24,67,76,77]. However, a recent study showed that different sub-genus oligotypes of *Prevotella* and *Bacteroides* are associated with plant-based and meat-based diets [23]. Das et al found that the *Prevotella* in the Indian samples mapped mainly to the animal-based diet-associated oligotypes [32]. However, >90% of the *Prevotella* in the Indian samples were unique and could not be fully mapped to the known plant- and animal-based diet-associated oligotypes [32]. In light of these studies, it would be an oversimplification to link our finding of greater *Prevotella* abundance in the Indian subjects in our study (as compared to subjects from other countries) with a more plant-rich diet or to link the greater abundance of *Prevotella* in Ladakh subjects as compared to Jaisalmer and Khargone with the former’s relatively higher consumption of animal protein and lower consumption of plant fiber.

*Bifidobacterium* was the second most abundant genus among all Indian subjects. *Bifidobacterium* abundance has previously been associated with consumption of dairy products in lactase non-persisters individuals in Western populations [78] and has been found to be completely absent in the traditional Hadza hunter-gatherers. It is likely that the high representation of *Bifidobacterium* in the Indian subjects is due to regular dairy consumption coupled with lactase non-persister status. In this regard, it is also interesting that we have found *Lactobacillus*, which also utilizes lactose as a major nutrient, to be significantly over-represented in Indian subjects as compared to subjects from other countries. *Bifidobacterium* and *Lactobacillus* abundance have also previously been shown to be high in the Indian Sikkim tribes whose diet is rich in diary and fermented foods [30].

Dietary intake shapes the gut community structure; hence, it is useful to pinpoint dietary changes that might be adopted to manipulate the gut microbial composition and thus alter the health status of a subject [4,74,79–82]. Indeed, a recent study revealed that genetic ancestry is not significantly associated with microbiome composition; on the contrary, healthy individuals with distinct ancestral origins who share a relatively common environment tend to have similar gut microbiota [83]. Identifying associations between a particular food in the diet and a particular genus is however not trivial, due to the complex and inter-dependent nature of the gut microbiome. The gut microbial communities derive their nutrition from carbohydrates [84] that come from host as well as dietary glycans [36,85]. The capacity of the gut flora to degrade carbohydrates is immense [86] and identifying accurate CAZyme information is therefore important. CAZymes were found to be almost similarly abundant in subjects from different geographical regions. This might be due to the stringent methodology we used to identify the true CAZymes in all the populations. Several differentially abundant CAZymes were identified that include carbohydrate esterases in subjects from Denmark and USA, and glycosyltransferases in subjects from Germany. A significant overlap was observed in between subjects from France and USA, suggesting that the sampled subjects might have shared a similar diet and lifestyle.

Although the CAZyme profile seems to be quite similar among the Indian regions studied, some differentially abundant CAZymes were present. For instance, a few CBMs were found to be differentially abundant in Jaisalmer subjects. Our correlation network study identified CAZymes/CBMS that are associated with specific genera in the different Indian geographical regions studied. The correlation networks are different in the different geographical regions, and interestingly, these interactions involve genera that are differentially abundant among regions but not necessarily very abundant. This suggests the role of less abundant genera in shaping the gut environment.

## Conclusions

In summary, our characterization of the fecal microbiota of healthy adult subjects of Indian ethnic tribes from Ladakh, Jaisalmer and Khargone presents a suite of unique features that suggest specific adaptation to a foraging lifestyle, with key genera *Prevotella* and *Bifidobacterium* contributing to the different taxonomic composition in these regions. The taxonomic composition of the fecal microbiota from Indian subjects is distinct as compared to subjects from other countries, with genera *Lactobacillus*, *Prevotella*, and *Bifidobacterium*, being more abundant, and *Bacteroides* being less abundant in Indian subjects and with variations in other genera of lower abundance. Even if there are taxonomic similarities between human populations, at finer scales their microbial communities may exhibit metabolic differences to suit dissimilar environmental constraints. The redundancy in CAZymes found in human gut indicates that activity, rather than composition, is conserved. Albeit, no unique CAZymes are present in the gut microbiota of subjects of different countries, their abundance is significantly different among different countries. The correlation networks of CAZymes and genus abundance in our study identified several CAZymes and carbohydrate-binding proteins that are associated with specific genera among the samples from Ladakh, Jaisalmer and Khargone. Further, the study of interactions of diet and CAZymes with taxonomy proved the supporting role of less abundant genera in shaping the activity in the human gut environment.

## Supporting information

S1 Fig Rarefaction curves representing microbial genera richness for samples sequenced in the study. X-axis represents the number of reads sampled from leaves and Y-axis represents the number of leaves (genera) in taxonomy. Each line represents one sample.
(TIF)

S2 Fig Heat maps showing relative abundance of microbial genera in the Indian regions. a) Heat map showing the relative abundance of the microbial genera >1% in at least one of the subjects from three Indian regions, obtained through One Codex. b) Heat map showing the relative abundance of the microbial genera >1% in at least one of the subjects from three Indian regions, obtained through MetaPhlAn. The ends of the color key represent the 5 and 95 quantile values. The values below 5 quantiles are dark red and values over 95 quantiles are dark blue.
(EPS)

S3 Fig Box plots representing differences in microbial taxa in the Indian regions. **a)** Box plot showing the relative abundance of microbial families with abundance greater than 1% in at least one sample in the subjects from Khargone, Jaisalmer, and Ladakh as obtained by Metaphlan (Green) and OneCodex (Blue) analysis. b) Box plot showing the relative abundance of the microbial genera with abundance greater than 1% in at least one sample in the subjects from Khargone, Jaisalmer, and Ladakh as obtained by Metaphlan (Green) and OneCodex (Blue) analysis.
(EPS)

S4 Fig Boxplots representing the differences in genus and phylum abundances by MetaPhlAn and OneCodex analysis in the Indian regions. a) *Prevotella* b) *Bifidobacterium* c) Bacteroidetes d) Actinobacteria **e)** Firmicutes. The multiple-group comparison is made using Kruskal-Wallis test and the P value for the testing is mentioned in the top of each plot. Welch t -test Bonferroni corrected P values are mentioned for pairwise comparisons. The values indicated in red are significant.
(EPS)

S5 Fig Abundance of microbial genera in subjects from different countries. **a)** Heat map showing the relative abundance of the microbial genera >1% in at least one of the subjects from different countries, obtained through MetaPhlAn. Box plot showing differences in b) Bacteroides c) Prevotella d) Lactobacillus e) Bifidobacterium abundance in different countries. The multiple-group comparison is made using Kruskal-Wallis test and the P value for the testing is mentioned in the top-left of each plot. Welch t -test Bonferroni corrected P values for pairwise comparisons are mentioned in a tabular format. The values indicated in red are significant.
(EPS)

S1 Table Metadata information of Indian samples. (XLSX)

S2 Table Detailed dietary information. The detailed dietary data for each participant is recorded as mentioned by the subjects.
(XLSX)

S3 Table Sample information for Indian subjects in this study. Information of samples, sequencing reads, preprocessing or reads, assembly, annotation and taxonomically assigned reads.
(XLSX)

S4 Table Taxonomic profiling. Genera, families and phyla identified in Indian samples by MetaPhlan and OneCodex analysis.
(XLSX)

S5 Table Genus abundance. Genus abundance significantly different within Indian regions and in between different countries.
(XLSX)

S6 Table Sample information for subjects from other countries. List of sample identifiers selected from the other country’s shotgun data.
(XLSX)

S7 Table Taxonomic profiling of samples from different countries. Genera, families and phyla identified in samples of different countries by MetaPhlan analysis.
(XLSX)

S8 Table CAZyme profiling. CAZymes identified in samples of Indian regions and different countries.
(XLSX)

S9 Table CAZyme abundance. CAZyme abundance significantly different within Indian regions and in between different countries.
(XLSX)
