# Correction: Metagenomics analysis reveals features unique to Indian distal gut microbiota

**DOI:** 10.1371/journal.pone.0243397

**Published:** 2020-12-01

**Authors:** The PLOS ONE Staff

The fifth author, T. N. C. Ramya, was incorrectly attributed equal contribution to this work with Kamaldeep Kaur, Indu Khatri, and Akil Akhtar. Only the first three authors, Kamaldeep Kaur, Indu Khatri, and Akil Akhtar, should be noted as contributing equally to this work. The publisher apologizes for the error.
