# Supplementary figures and images for: Metagenomics analysis reveals features unique to Indian distal gut microbiota
Source: PLoS One. 2020 Apr 8;15(4):e0231197. doi: 10.1371/journal.pone.0231197 (PMC7141701; doi:10.1371/journal.pone.0231197)

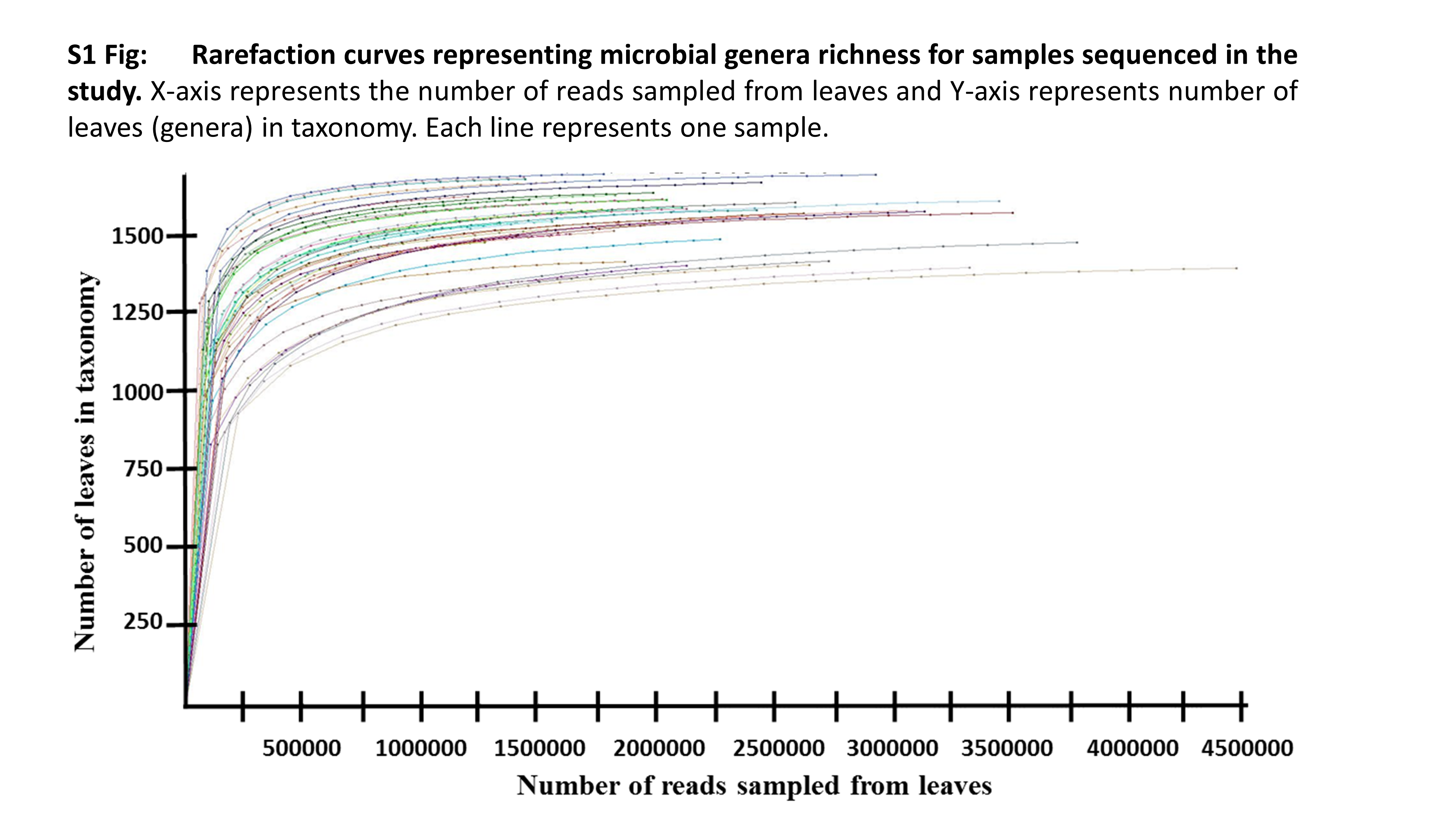

Supplement: S1 Fig — X-axis represents the number of reads sampled from leaves and Y-axis represents the number of leaves (genera) in taxonomy. Each line represents one sample. (TIF) [file pone.0231197.s001.tif]
